# Premature Burial

**Published:** 1904-03-12

**Authors:** J. R. Williamson


### EDITOR'S LETTER-BOX.

**[Our Correspondents are reminded that prolixity is a great bar to publication, and that brevity of style and conciseness of statement greatly facilitate early insertion.]**

### PREMATURE BURIAL.

MR. J. R. WILLIAMSON writes: You have performed a great service in the interests of humanity by the sensible remarks on this important question in THE HOSPITAL of 26th inst., and I beg to thank you on behalf of the London Association for the Prevention of Premature Burial. One of those cases which occur from time to time, and prove the necessity for speedy reform in the present system of death certification, in order to prevent, not only burial alive, but fraud on the part of unprincipled persons, was the cause of a criminal charge being made against a woman at the Glamorgan Assizes last July. It appeared from the evidence of a doctor that on April 16th a woman came to him with a child (then in Court), and the doctor on examination found that it was suffering from measles and bronchitis. On the following day the mother called and reported that the child was dead, whereupon, without taking the precaution to see and examine the body, the medical man accepted the *ipse dixit* of the woman, and promptly gave her a certificate of death, as she said, "in the usual way." The Judge, addressing the doctor, said: "I should not have thought that the universal custom of the profession was to certify the death of a child which you had not seen. There are now exceptional opportunities in this town of ascertaining if that is the professional custom—the Medical Association will tell us no doubt." The Judge further remarked: "I am bound to say I cannot accept your (the doctor's) views, and am quite sure, if this custom is universal, it is nevertheless wrong. I don't suggest against you that you did anything wrong except to accept a woman's statement which turned out to be untrue. Many men have done that before. If you have seen the child once and are told it was subsequently dead, I don't think you are justified in certifying it died from the disease from which you happen to know it is suffering. A little more caution is necessary." The woman was found guilty of obtaining £3 10s. from a friendly society by pretending that her child had died at Swansea on June 17th, and sentenced to six months' imprisonment. The Bill drafted by the above association would put an end to this state of affairs, and also prevent living children being certified as stillborn, as it provides (to put it as briefly as may be): (1) That no burial shall take place without a medical certificate of death; (2) that no such certificate shall be given without personal examinations of the alleged deceased, in order to ascertain that putrefactive decomposition, the only sure proof of dissolution, has unmistakably set in; (3) that if any body be buried contrary to the provisions of the Bill, justices at petty sessions may issue an order for its exhumation; (4) that local authorities should provide waiting mortuaries, where bodies may be detained until the fact of death is ascertained beyond the shadow of doubt.

